# Geriatric Nutritional Risk Index is Associated with Hospital Death in Elderly Patients with Multiple Organ Dysfunction Syndrome: A Retrospective Study Based on the MIMIC-III Database

**DOI:** 10.3389/fnut.2022.834256

**Published:** 2022-06-03

**Authors:** Zhi Mao, Tao Wen, Xiaoli Liu, Jinsong Chen, Pan Hu, Chao Liu, Hui Liu, Hongjun Kang, Zhengbo Zhang, Feihu Zhou

**Affiliations:** ^1^Department of Critical Care Medicine, The First Medical Center, Chinese People's Liberation Army General Hospital, Beijing, China; ^2^School of Biological Science and Medical Engineering, Beihang University, Beijing, China; ^3^Department of Gerentology, Zhangzhou Zhengxing Geriatrics Hospital, Zhangzhou, China; ^4^Center for Artificial Intelligence in Medicine, Chinese Peoples Liberation Army General Hospital, Beijing, China; ^5^Department of Biomedical Engineering, Chinese People's Liberation Army General Hospital, Beijing, China

**Keywords:** elderly, geriatric nutritional risk index, multiple organ dysfunction syndrome, intensive care unit, MIMIC-III

## Abstract

**Purpose:**

Elderly patients with multiple organ dysfunction syndrome (MODS) have a higher mortality during hospitalization in the intensive care unit (ICU). Elderly patients often suffer from malnutrition. On the basis of the MIMIC-III database, this study analyzed the effect of the baseline nutritional status on the death of elderly patients with MODS during hospitalization.

**Materials and Methods:**

Elderly patients with MODS were screened out from MIMIC-III 1.4 database. The geriatric nutritional risk index (GNRI) was calculated and used to group patients into: normal nutrition (GNRI > 98) and malnutrition (GNRI ≤ 98) groups. The malnutrition group was divided into mild (92–98), moderate (82–91), and severe (≤81) groups. The differences in the baseline data and the incidence of adverse events between groups were compared. The GAM model was used to determine whether a curve relationship was present between the hospital death of elderly patients with MODS and GNRI and analyze the threshold saturation effect. The multivariate logistic regression was used to calculate the odds ratio (OR) of in-hospital deaths in different GNRI groups. The interaction test was performed to find subgroups with differences.

**Results:**

A total of 2456 elderly patients with MODS were enrolled. A total of 1,273 (51.8%) and 1183 (48.2%) patients were in the normal nutrition and malnutrition groups, respectively. The mortality rate of patients in the normal nutrition group during hospitalization was lower than that in the malnutrition group (206/1273 vs. 292/1183, X2 = 27.410, *P* < 0.001; OR = 0.59, 95% CI: 0.48–0.72). The GAM model fitting analysis showed a threshold saturation effect at GNRI = 92. Adjusted OR values with GNRI ≥ 92 began to change to 1, and GNRI and death had no association. At GNRI < 92, high GNRI related to low risk of death. Subgroup analysis of patients with GNRI < 92 showed that the risk of death in elderly male patients was lower than that of female patients.

**Conclusion:**

GNRI is related to the severity of illness in elderly patients with MODS. At GNRI < 92, moderate to severe malnutrition increases the risk of death in elderly patients with MODS during hospitalization.

## Introduction

With the extension of human life span, the number of elderly patients is increasing, and the number of elderly patients admitted to the intensive care unit (ICU) also increases (1). The mortality rate of patients in the ICU during hospitalization is significantly higher than that in the general ward (2). Elderly critically ill patients often have multiple diseases due to a variety of reasons, and their important organ functions have suffered age-related decline. Therefore, most elderly patients admitted to the ICU have multiple organ dysfunction syndrome (MODS). In many countries, the willingness of elderly patients and their families to treat is relatively weaker than that of non-elderly patients, which further causes the mortality rate of elderly critically ill patients to be significantly higher than that of non-elderly critically ill patients (3). The increase in the number of elderly critically ill patients and high mortality rate have increased the burden on the ICU and triggered discussion on the benefits of treatment of elderly patients in the ICU (1). After some elderly patients with MODS are admitted to the ICU, their conditions improve or are cured after treatment. However, many elderly patients with MODS are in their final stage of life, and significant results especially in those who suffer from malnutrition due to disease are difficult to achieve with active treatment (4).

Malnutrition has an adverse effect on the long-term prognosis of patients and has a high incidence in the elderly due to various comorbidities, strict dietary control and decreased appetite. Malnutrition is common in patients with chronic heart failure, renal failure, respiratory failure, and long-term consumption of malignant tumors (5). However, studies on the relationship between the nutritional status of elderly patients with MODS and the condition and treatment effect during hospitalization are few. The geriatric nutritional risk index (GNRI) is specifically used to evaluate the nutritional status of elderly patients (6, 7). A number of studies showed that this index can predict the short- and long-term prognoses of elderly patients. Moreover, the calculation of this index only requires height, weight, and serum albumin (ALB) level, which is simple to calculate, easy to obtain, and does not increase the additional medical expenditures of patients especially critically ill patients (8).

The MIMIC database provides a large number of real-world clinical data of critically ill patients. In recent years, this database has been deeply excavated and has produced many results with clinical application value (9). The purpose of this study is to analyze the relationship between the baseline nutritional status of elderly patients with MODS and their condition when admitted to the ICU and adverse events during hospitalization based on the MIMIC-III database.

## Materials and Methods

### Study Population

The subjects of this study are elderly patients with MODS admitted to the ICU recorded in the MIMIC-III database. The inclusion criteria are as follows: (1) age ≥ 65 years; (2) first admission to ICU (for elderly patients admitted to the ICU multiple times, only the first data are collected); (3) retention time in ICU ≥ 24 h; (4) sequential organ failure (SOFA) score ≥ 2 points and involvement of at least two organs; and (5) HR, RR, MAP, GCS, T, and SPO_2_ are measured at least once. The exclusion criteria are as follows: (1) loss of key data, such as the first SOFA and the first simplified acute physiology score I and (2) loss to follow-up. All patient-related information in the MIMIC-III database is anonymous, and no informed consent is required.

### Data Source

The data of this study come from the MIMIC-III database, an open critical medicine database jointly released by the Massachusetts Institute of Technology Computational Physiology Laboratory, Beth Israel Dikon Medical Center, and Philips Medical under the funding of the National Institutes of Health. The latest version is the MIMIC-III v1.4. This database collects the hospitalization information of more than 50,000 patients admitted to the ICU of Beth Israel Dikang Medical Center in the United States from 2002 to 2012. Data include vital signs, medications, laboratory measurements, observations, records drawn by nursing staff, fluid balance, surgical codes, diagnostic codes, imaging reports, hospital stay, and survival data. This database has passed ethical review and obtained right for use.

### Data Extraction

The PostgreSQL 10.7 software and SQL language are used to extract the following data from the MIMIC-III database: age, gender, weight, white blood cell count, hemoglobin, platelet count, blood urea nitrogen, blood creatinine, blood glucose, blood electrolytes (i.e., K^+^, Na^+^, and Ca^2+^), blood HCO^3−^, SOFA score, ICU hospital stay, death in the ICU, and other data. Complications (e.g., hypertension, coronary heart disease, chronic obstructive pulmonary disease, and chronic kidney disease); use of mechanical ventilation, continuous renal replacement therapy during ICU hospitalization. All laboratory test parameters are extracted from the data generated within the first 24 h after the patient enters the ICU (i.e., baseline value) and extreme values during the ICU hospitalization period (i.e., maximum and minimum values).

### Group

Groups are based on the GNRI. GNRI is calculated on the basis of height (m), weight (kg), and ALB level (g/L). The formula is: GNRI = 1.489 × ALB + 41.7 × [weight/(22 × height^2^)]. GNRI > 98 indicates normal nutritional status, and GNRI ≤ 98 means malnutrition (GNRI = 92–98, mild malnutrition; GNRI = 82–91, moderate malnutrition, and GNRI ≤ 81, severe malnutrition).

### Outcome

The endpoint observed in this study is the mortality rate within 28 days after the patient is admitted to the ICU.

### Statistical Analysis

Continuous variables are represented by the median [quartile, median (Q1–Q3)]. The Wilcoxon rank–sum test is used to compare the two groups, and the Kruskal–Wallis test is used for comparison between groups. Categorical variables are expressed as percentages (%), and comparisons between groups are performed using the χ^2^-test. The generalized additive model (GAM) is used to fit the curve relationship between in-hospital death of elderly patients with MODS and GNRI, and the threshold saturation effect is analyzed. The multivariate logistic regression equation is used to calculate the odds ratio (OR) of in-hospital deaths among different groups. *P* < 0.05 indicates that the difference is statistically significant. The statistical analysis of all data is done using the EmpowerStats (http://www.empowerstats.com, version 3.4.3 R software package) software.

## Results

### Baseline Characteristics

**Figure 1:**
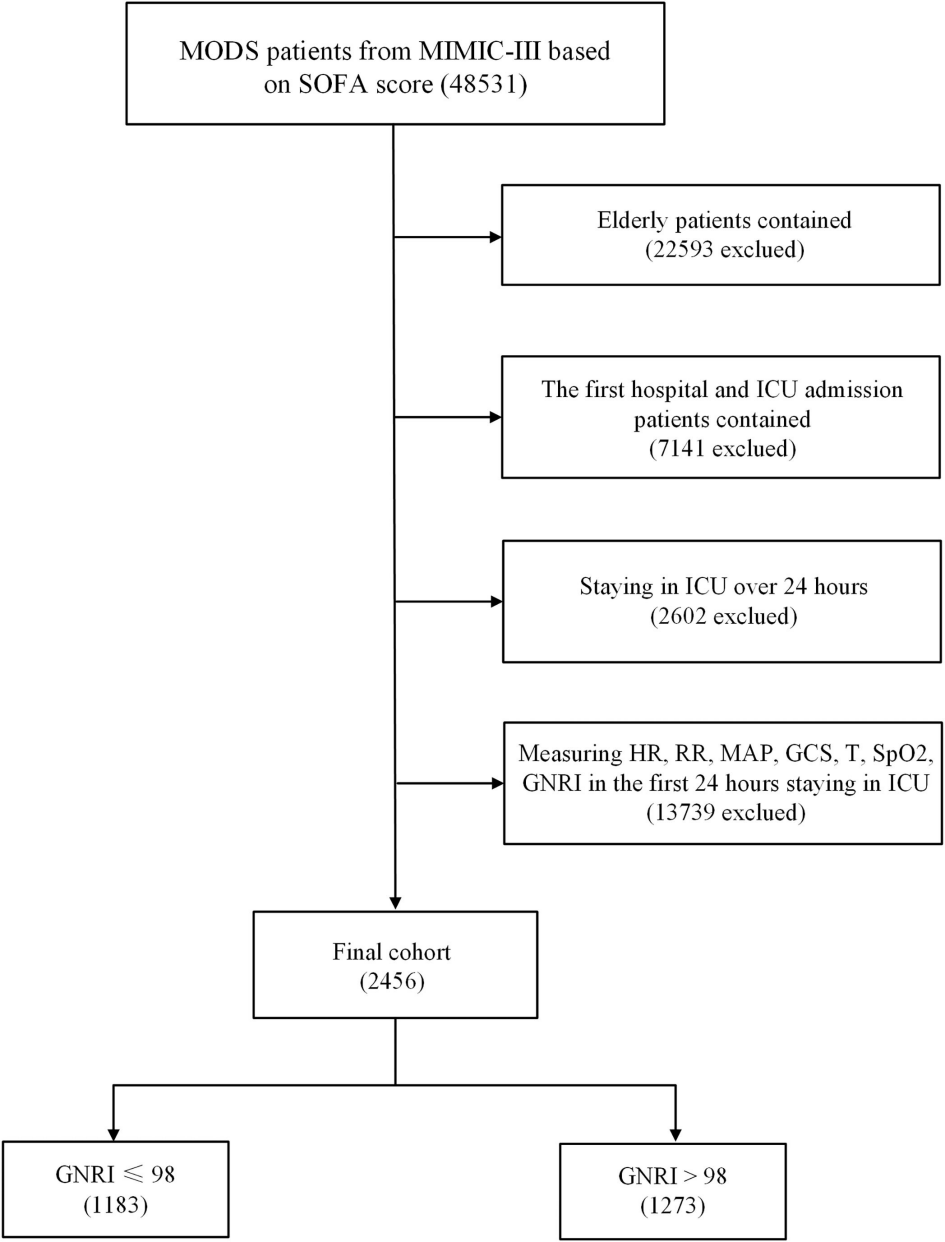
Flowchart of patient selection.

After screening, 2,456 elderly critically ill patients were included (Figure 1). Patients' age ranged from 65 to 91 years old with a median age of 77 years (71, 83). A total of 1135 (46.2%) and 1321 (53.8%) patients were males and females, respectively. The GNRI of all patients was 53–170, and the median GNRI was 99 (89, 109). In accordance with GNRI, patients were preliminarily divided into normal nutrition (GNRI > 98, 1273 cases, 51.8%) and malnutrition (GNRI ≤ 98, 1183 cases, 48.2%) groups. The malnutrition group was further divided into mild (GNRI = 92–98, 444 cases), moderate (GNRI = 82–91, 480 cases), and severe (GNRI ≤ 81, 259 cases) malnutrition groups. The baseline data comparison of each group is shown in Tables 1, 2.

**Table 1:** Characteristics of patients in the normal nutrition and malnutrition groups.

**Characteristics**	**Total**	**GNRI > 98**	**GNRI ≤98**	***P*-value**
*N*	2,456	1,273	1,183	
Age (years)	77 (71, 83)	76 (70, 81)	79 (73, 84)	<0.001
Male, *n* (%)	1135 (46.2%)	697 (54.8)	624 (52.7)	0.319
Ethnicity, *n* (%)				0.018
Asian	53 (2.16%)	13 (1.0)	40 (3.4)	
Black	155 (6.31%)	85 (6.7)	70 (5.9)	
Hispanic	35 (1.43%)	17 (1.4)	18 (1.5)	
White	1790 (72.88%)	950 (74.6)	840 (71.0)	
Other	423 (17.22%)	208 (16.3)	215 (18.2)	
BMI (kg/m^2^)	27.3 (23.8, 31.5)	30.8 (27.2, 35.2)	24.1 (21.6, 27.0)	<0.001
Hospital days	10 (6, 17)	9 (6, 15)	11 (6, 19)	<0.001
ICU days	4 (2, 8)	4 (2, 8)	4 (2, 9)	0.175
SPO_2_ (%)	98 (96, 100)	98 (95,100)	98 (96,100)	0.12
Heart rate (/min)	85 (73, 100)	87 (75, 103)	83 (71, 97)	<0.001
Respiratory rate (/min)	18 (15, 23)	18 (15, 23)	19 (15, 23)	0.017
MAP (mmHg)	78 (67, 91)	80 (69, 93)	75.0 (65, 89)	<0.001
Temperature (°C)	36.6 (36.0-37.1)	36.6 (36.0, 37.1)	36.5 (35.9, 37.1)	0.176
First Care Unit, *n* (%)				<0.001
CCU	678 (27.61%)	402 (31.58%)	276 (23.3%)	
CSRU	349 (14.21%)	207 (16.26%)	142 (12%)	
MICU	919 (37.42%)	410 (32.21%)	509 (43%)	
SICU	318 (12.95%)	163 (12.80%)	155 (13.1%)	
TSICU	192 (7.82%)	91 (7.15%)	101 (8.5%)	
**Comorbidities**, ***n*** **(%)**				
CLF	115 (4.68%)	63 (4.95%)	52 (4.4%)	0.58
ALF	105 (4.28%)	50 (3.93%)	55 (4.6%)	0.433
COPD	587 (23.90%)	295 (23.17%)	292 (24.7%)	0.407
ARDS	697 (28.38%)	329 (25.84%)	368 (31.1%)	0.004
CAD	1195 (48.66%)	663 (52.08%)	532 (45%)	<0.001
AKI	949 (38.64%)	476 (37.39%)	473 (40%)	0.202
AHF	323 (13.15%)	204 (16.03%)	119 (10.1%)	<0.001
Stroke	155 (6.31%)	86 (6.76%)	69 (5.8%)	0.391
CHF	1106 (45.03%)	565 (44.38%)	541 (45.7%)	0.529
CRF	384 (15.64%)	217 (17.05%)	167 (14.1%)	0.052
Malignancy	201 (8.18%)	86 (6.76%)	115 (9.7%)	0.009
**Scoring systems**				
GCS	15 (15, 15)	15 (15, 15)	15 (15, 15)	0.057
Shock index	0.72 (0.57, 0.89)	0.68 (0.54, 0.84)	0.76 (0.60, 0.93)	<0.001
SOFA first day	5 (3, 8)	5 (3, 7)	5 (3, 8)	<0.001
APSIII first day	49 (37, 63)	46 (35, 60)	52 (41, 67)	<0.001

*GNRI, geriatric nutritional risk index; CCU, cardiac care unit; CSRU, cardiac surgical care unit; MICU, medical intensive care unit; SICU, surgical intensive care unit; TSICU, trauma surgical intensive care unit; CLF, chronic liver failure; ALF, acute liver failure; COPD, chronic obstructive pulmonary disease; ARDS, acute respiratory distress syndrome; CAD, coronary artery disease; AKI, acute kidney injury; AHF, acute heart failure; CHF, chronic heart failure; CRF, chronic renal failure; GCS, Glasgow score; SOFA, Sequential organ failure assessment; APSu, acute physiology score III*.

**Table 2:** Characteristics of patients in subgroups with different GNRIs.

**Characteristics**	**GNRI**				***P*-value**
	**≤81 (*n* = 259)**	**82–91 (*n* = 480)**	**92–98 (*n* = 444)**	**>98 (*n* = 1,273)**	
Age (years)	78 (73, 84)	70 (72, 85)	79(73, 84)	76 (70, 81)	<0.001
Male	124 (47.9)	256 (53.3)	244 (55.0)	697 (54.8)	0.221
Ethnicity, *n* (%)					0.018
Asian	12 (4.63%)	12 (2.50%)	16 (3.60%)	13 (1.02%)	
Black	16 (6.18%)	27 (5.62%)	27 (6.08%)	85 (6.68%)	
Hispanic	3 (1.16%)	9 (1.88%)	6 (1.35%)	17 (1.34%)	
White	177 (68.34%)	344 (71.67%)	319 (71.85%)	950 (74.63%)	
Other	51 (19.69%)	88 (18.33%)	76 (17.12%)	208 (16.34%)	
BMI (kg/m^2^)	21.4 (19.3, 24.1)	24.2 (21.7, 26.9)	25.7 (23.0, 28.5)	30.8 (27.2, 35.2)	<0.001
First care unit					<0.001
CCU	43 (16.60%)	106 (22.08%)	127 (28.60%)	402 (31.58%)	
CSRU	23 (8.88%)	61 (12.71%)	58 (13.06%)	207 (16.26%)	
MICU	127 (49.03%)	207 (43.12%)	175 (39.41%)	410 (32.21%)	
SICU	42 (16.22%)	58 (12.08%)	55 (12.39%)	163 (12.80%)	
TSICU	24 (9.27%)	48 (10.00%)	29 (6.53%)	91 (7.15%)	
SPO_2_ (%)	99 (96, 100)	98.0 (96, 100)	98.0 (96, 100)	98.0 (95, 100)	0.025
Heart rate (/min)	90.0 (79, 109)	86.0 (75, 100)	85.0 (72, 103)	83.0 (71, 97)	<0.001
Respiratory rate (/min)	19.0 (15, 25)	19 (14, 22)	19.0 (15, 23)	18.0 (15, 23)	0.066
Temperature (°C)	36.4 (35.7, 37.1)	36.5 (35.9, 37.1)	36.6 (36.0, 37.1)	36.6 (36.0, 37.1)	0.118
SBP (mmHg)	110 (97, 131)	116 (99, 134)	119 (101, 138)	123 (105, 141)	<0.001
DBP (mmHg)	56 (47, 67)	56 (47, 66)	60 (50, 72)	61 (51, 72)	<0.001
MBP (mmHg)	74 (63, 87)	75 (64, 88)	77 (66, 92)	80 (69, 93)	<0.001
**Laboratory parameters**					
ALB (g/dL)	2.3 (1.9, 2.6)	2.7 (2.4, 3.1)	3.1 (2.8, 3.4)	3.5 (3.1, 3.8)	<0.001
ALT (U/L)	27 (16.0, 42.0)	27 (19.0, 51.8)	27 (17.0, 49.0)	27 (18.0, 45.0)	0.098
AST (U/L)	39 (24.50, 63.5)	39 (28.0, 75.0)	39 (28.75, 77.3)	39 (25.00, 69.0)	0.4
ALP (U/L)	79 (68.0, 139.5)	79 (59.0, 117.3)	79 (63.0, 105.3)	79 (61.0, 100.0)	<0.001
Bilirubin (mg/dL)	0.6 (0.40, 1.20)	0.6 (0.40, 1.00)	0.6 (0.50, 0.92)	0.6 (0.50, 1.00)	0.564
BUN (mg/dL)	27 (17.0, 46.5)	29 (19.0, 44.3)	30 (19.0, 44.0)	26 (18.0, 43.0)	0.898
CR (mg/dL)	1.1 (0.70, 1.70)	1.2 (0.90, 2.00)	1.3 (0.90, 2.00)	1.2 (0.90, 1.90)	0.188
Potassium (mmol/L)	4.1 (3.70, 4.75)	4.2 (3.70, 4.70)	4.2 (3.80, 4.70)	4.2 (3.80, 4.70)	0.777
Sodium (mmol/L)	138 (134, 141)	138 (135, 141)	138 (135, 141)	138 (135, 141)	0.713
Chloride (mmol/L)	105 (101, 109)	105 (100, 109)	104 (100, 108)	103 (99, 106)	<0.001
Magnesium (mmol/L)	1.9 (1.60, 2.20)	1.9 (1.60, 2.20)	1.9 (1.70, 2.20)	2 (1.80, 2.20)	0.149
Bicarbonate (mmol/L)	22.0 (19.0, 26.0)	22.0 (19.0, 26.0)	22.0 (20.0, 26.0)	23.0 (20.0, 27.0)	<0.001
Glucose (mmol/L)	128 (101, 157)	132 (107, 169)	135 (111, 182)	142 (114, 192)	<0.001
HCT (%)	31.9 (28.0, 36.5)	32 (28.6, 36.3)	33.35 (29.7, 37.0)	34.6 (30.4, 38.5)	<0.001
HGB (g/dL)	10.3 (9.2, 12.1)	10.7 (9.5, 12.0)	11.2 (9.8, 12.5)	11.6 (10.2, 13.0)	<0.001
Platelet (1,000/mm3)	225 (138, 317)	215 (144, 304)	210 (156, 281)	216 (160, 282)	0.111
PT (second)	14.7 (13.6, 16.9)	14.3 (13.3, 16.5)	14.3 (13.3, 16.8)	14.3 (13.1, 16.7)	0.8
APTT (second)	33.9 (29.4, 43.1)	32.3 (27.4, 42.1)	32.3 (28.0, 41.8)	32 (26.6, 43.1)	0.92
INR	1.3 (1.20, 1.70)	1.3 (1.20, 1.60)	1.3 (1.20, 1.60)	1.3 (1.10, 1.60)	0.712
WBC (10^9^/L)	12.9 (8.6, 17.9)	11.9 (8.2, 16.4)	12 (8.4, 15.7)	10.9 (7.8, 15.6)	0.558
**Comorbidities**, ***n*** **(%)**					
CLF	14 (5.41%)	23 (4.79%)	15 (3.38%)	63 (4.95%)	0.53
ALF	14 (5.41%)	25 (5.21%)	16 (3.60%)	50 (3.93%)	0.441
COPD	71 (27.41%)	117 (24.38%)	104 (23.42%)	295 (23.17%)	0.524
ARDS	97 (37.45%)	149 (31.04%)	122 (27.48%)	329 (25.84%)	<0.001
CAD	96 (37.07%)	216 (45.00%)	220 (49.55%)	663 (52.08%)	<0.001
AKI	99 (38.22%)	190 (39.58%)	184 (41.44%)	476 (37.39%)	0.474
AHF	19 (7.34%)	45 (9.38%)	55 (12.39%)	204 (16.03%)	<0.001
Stroke	9 (3.47%)	27 (5.62%)	33 (7.43%)	86 (6.76%)	0.153
CHF	105 (40.54%)	218 (45.42%)	218 (49.10%)	565 (44.38%)	0.15
CRF	21 (8.11%)	77 (16.04%)	69 (15.54%)	217 (17.05%)	0.004
Malignancy	31 (11.97%)	51 (10.62%)	33 (7.43%)	86 (6.76%)	0.006
**Scoring systems**					
GCS	15 (15, 15)	15 (15, 15)	15(15, 15)	15 (15, 15)	0.123
Shock index	0.8 (0.7, 1.0)	0.8 (0.6, 0.9)	0.8 (0.6–0.9)	0.7 (0.5, 0.8)	<0.001
SOFA first day	6 (4, 8)	5 (3, 8)	5 (3, 7)	5 (3, 7)	<0.001
APSIII first day	60 (48, 74)	53 (41, 66)	48 (38, 61)	46 (35, 60)	<0.001

*GNRI, geriatric nutritional risk index; CCU, cardiac care unit; CSRU, cardiac surgical care unit; MICU, medical intensive care unit; SICU, surgical intensive care unit; TSICU, trauma surgical intensive care unit; SBP, systolic blood pressure; DBP, diastolic blood pressure; ALB, albumin; ALT, alanine transaminase; AST, aspartate aminotransferase; ALP, a lkaline phosphatase; CR, creatine; BUN, blood urea nitrogen; HCT, hematocrit; HGB, hemoglobin; PT, prothrombin time; APTT, activated partial thromboplastin time; WBC, white blood cell; INR, international normalized ratio; CLF, chronic liver failure; ALF, acute liver failure; COPD, chronic obstructive pulmonary disease; ARDS, acute respiratory distress syndrome; CAD, coronary artery disease; AKI, acute kidney injury; AHF, acute heart failure; CHF, chronic heart failure; CRF, chronic renal failure; GCS, Glasgow score; SOFA, Sequential organ failure assessment; APSu, acute physiology score III*.

### Relationship Between Baseline Nutritional Status and Illness Severity

Among all patients, 1,875 had acute kidney injury, and all patients had sepsis. A total of 2042 (83.1%) patients had a GCS score of 15, a shock index of 0.26–2.94 with a median of 0.72 (0.57, 0.89), and SOFA score of 2–21 with a median of 5 (3, 8). A total of 1496 patients received mechanical ventilation, and 127 patients received CRRT. The comparison between each group is shown in Tables 3, 4.

**Table 3:** Comparison of the conditions of patients in the normal and malnutrition groups.

**Indicators**	**GNRI > 98 (*n* = 1,273)**	**GNRI ≤98 (*n* = 1,183)**	***P*-value**
AKI	999 (78.5)	876 (74.0)	0.010
SEPSIS	1273 (100.0)	1183 (100.0)	1.000
GCS	15 (15, 15)	15 (15, 15)	<0.001
SI	0.68 (0.54, 0.84)	0.76 (0.60, 0.93)	<0.001
SOFA	5 (3, 7)	5 (3, 8)	<0.001
CRRT	70 (5.5)	56 (4.7)	0.391
Ventilation	789 (62.0)	706 (59.7)	0.243

*GNRI, geriatric nutritional risk index; AKI, acute kidney injury; GCS, Glasgow score; SI, shock index; SOFA, Sequential organ failure assessment; CRRT, continuous renal replacement therapy*.

**Table 4:** Comparison of important indicators among the four subgroups.

**Characteristics**	**GNRI**				***P*-value**
	**>98 (*n* = 1,273)**	**92–98 (*n* = 444)**	**82–91 (*n* = 480)**	**≤81 (*n* = 259)**	
AKI	999 (78.5)	332 (74.8)	354 (73.8)	190 (73.4)	0.076
SEPSIS	1273 (100.0)	444 (100.0)	480 (100.0)	259 (100.0)	1.000
GCS	15 (15, 15)	15 (15, 15)	15 (15, 15)	15 (15, 15)	0.582
SI	0.68 (0.54, 0.84)	0.75 (0.58, 0.91)	0.75 (0.61, 0.92)	0.81 (0.66, 0.99)	<0.001
SOFA	5 (3, 7)	5 (3, 7)	5 (3, 8)	6 (4, 8)	<0.001
CRRT	70 (5.5)	21 (4.7)	22 (4.6)	13 (5.0)	0.849
Ventilation	789 (62.0)	248 (55.9)	288 (60.0)	170 (65.6)	0.047

*GNRI, geriatric nutritional risk index; AKI, acute kidney injury; GCS, Glasgow score; SI, shock index; SOFA, Sequential organ failure assessment; CRRT, continuous renal replacement therapy*.

### Hospital Outcome

Among all patients, 498 died during hospitalization. The median length of hospitalization was 10 (6, 17) days, and the median length of ICU hospitalization was 4 (2, 8) days. The mortality, length of stay in hospital, and ICU stay in the normal nutrition group were lower than those in the malnutrition group (Table 5). Subgroup analysis showed differences in mortality during hospitalization and ICU stay time between groups, but no statistical difference in hospitalization time was observed (Table 6).

**Table 5:** Comparison of hospitalization outcomes and prognosis of patients in malnutrition and normal nutrition groups.

**Indicators**	**GNRI > 98 (*n* = 1,273)**	**GNRI ≤98 (*n* = 1,183)**	***P*-value**
Hospital death	206 (16.2)	292 (24.7)	<0.001
Hospital duration	9 (6, 15)	11 (6, 19)	<0.001
ICU duration	4 (2, 8)	4 (2, 9)	<0.001

*GNRI, geriatric nutritional risk index; ICU, intensive care unit*.

**Table 6:** Comparison of hospitalization outcomes and prognosis of patients with different nutritional status.

**Characteristics**	**GNRI**				***P*-value**
	**>98 (*n* = 1,273)**	**92–98 (*n* = 444)**	**82–91 (*n* = 480)**	**≤81 (*n* = 259)**	
Hospital death	206 (16.2)	89 (20.1)	115 (24.0)	88 (34.0)	<0.001
Hospital duration	9 (6, 16)	10 (6, 17)	10.0 (6.4–17.8)	12.9 (7.6–22.5)	<0.001
ICU duration	4 (2, 8)	4 (2, 8)	4(2, 8)	5 (2, 11)	0.354

*GNRI, geriatric nutritional risk index; ICU, intensive care unit*.

### Relationship Between GNRI and Death During Hospitalization of Elderly Critically Ill Patients

**Figure 2:**
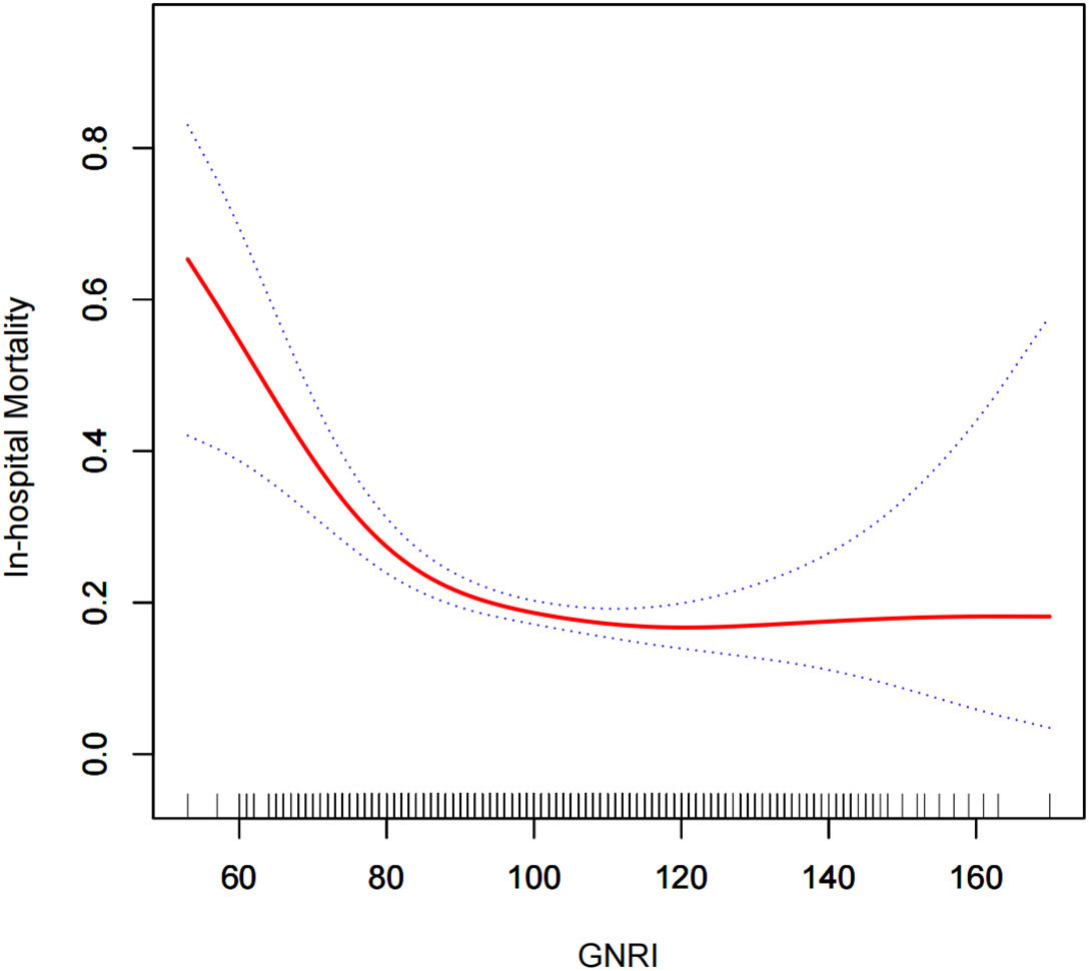
Relationship between GNRI and death during hospitalization. The curve fitting equations of death (Y) and GNRI (X) during hospitalization are used. A non-linear relationship is observed between Y and X, and the slope changes evidently, which may have a threshold saturation effect.

A total of 206 (16.2%) and 292 (24.7%) patients died during hospitalization in the normal nutrition and malnutrition groups, respectively. Malnutrition significantly increased the mortality risk of elderly patients with MODS during hospitalization (OR = 1.70, 95% CI: 1.39–2.07). After adjusting for confounding factors, a curve fitting equation was made for death during hospitalization and GNRI by using the GAM model. A non-linear relationship between death during hospitalization and GNRI was observed, and the slope changed significantly, which might have a threshold saturation effect (Figure 2). The threshold effect analysis was applied to find the inflection point, and changes in OR between the two groups before and after the inflection point were compared. Further analysis showed a threshold saturation effect when GNRI = 92, that is, as the GNRI increased to 92, a group of adjusted OR values with GNRI ≥ 92 began to change to 1. No correlation was observed between death during hospitalization and GNRI.

**Figure 3:**
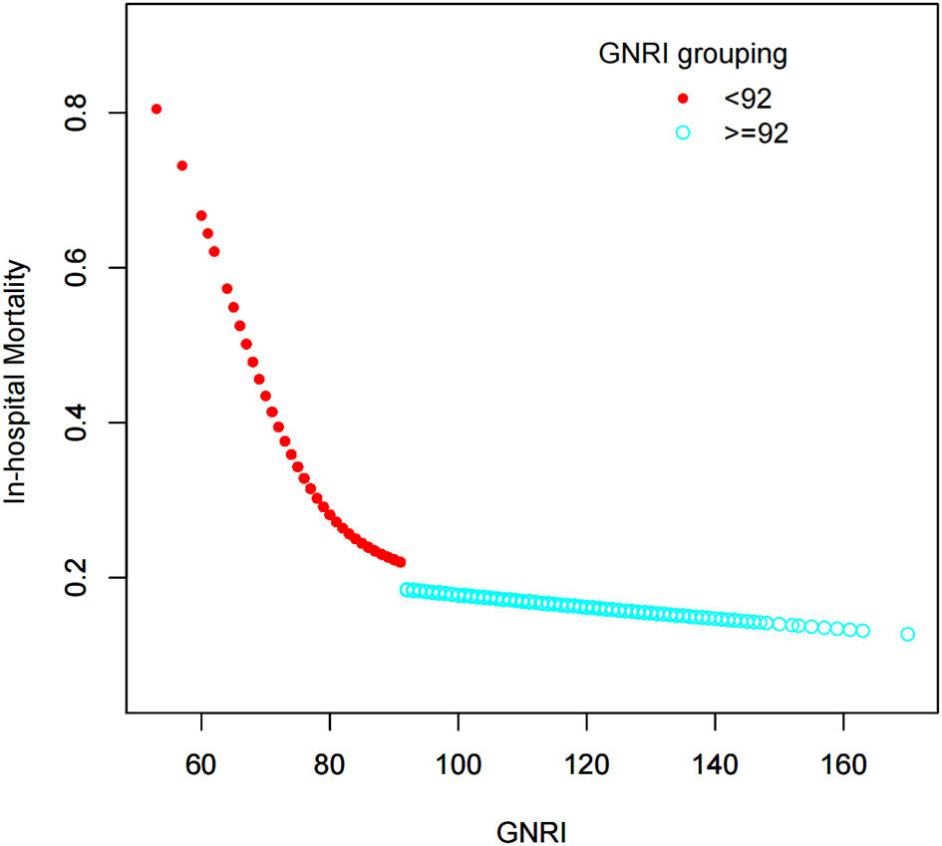
Relationship between GNRI and death during hospitalization. In accordance with the GNRI cutoff of 92, the curve fitting equation is performed again. Evident differences can be observed in the slopes of the two equations before and after the cutoff value.

**Figure 4:**
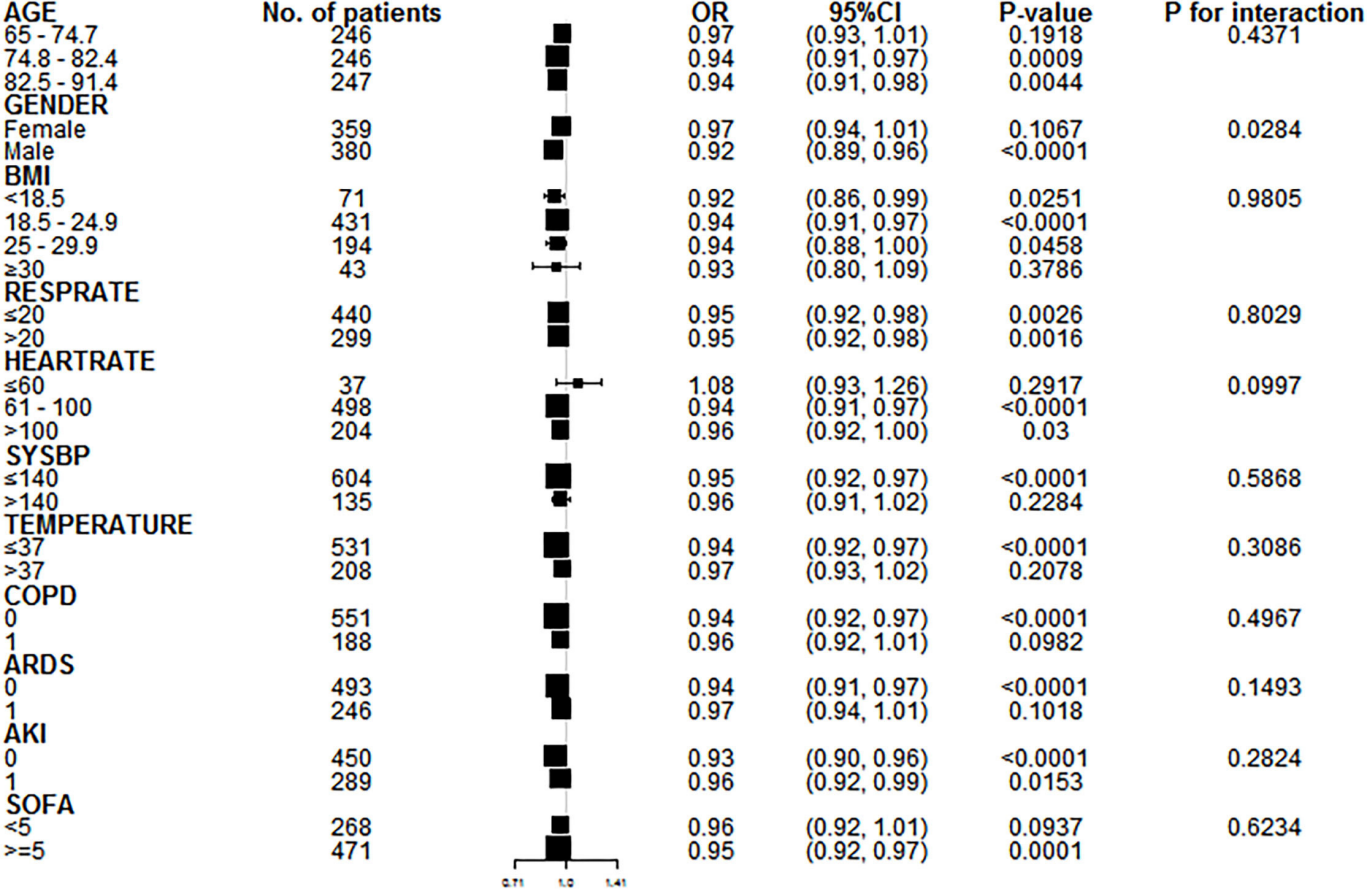
Subgroup analysis of patients with GNRI < 92. A further subgroup analysis of 739 elderly patients with GNRI < 92 is performed to verify the consistency of the relationship between Y and X. Except gender, all other variables have no interaction (*P* for interaction > 0.05). In the results of gender stratification, the risk of death for older men is lower than that for older women.

Compared with patients with GNRI ≥ 92, moderate to severe malnutrition (GNRI < 92) further increased the mortality of elderly severely ill patients during hospitalization (OR = 1.83, 95% CI: 1.49–2.24, *P* < 0.001; Table 7). According to the tangent point value of 92, the curve fitting equation was performed again, and the slopes of the two equations before and after the tangent point were evidently different (Figure 3). A total of 739 patients with GNRI < 92 were extracted, and the multiple regression equation was applied. The adjusted OR and interval were 0.94 (0.92, 0.97), indicating that GNRI was a protective factor for severe patients with GNRI < 92. Moreover, the moderate-risk group (82–91) had a lower rate of death than the high-risk group (<82). Thus, a large GNRI resulted in low risk of death (Table 8). A further subgroup analysis was performed on 739 elderly patients with GNRI < 92 to verify the consistency of the relationship between death during hospitalization and GNRI. Except gender, all other variables had no interaction (*P* for interaction > 0.05). In the results of gender stratification, the risk of death for older men was lower than that for older women (Figure 4).

**Table 7:** Threshold effect analysis of GNRI on in-hospital mortality.

	**Non-adjusted**		**Adjust model-I**		**Adjust model-II**	
	**OR**	***P***	**OR**	***P***	**OR**	***P***
Total	0.98 (0.97, 0.99)	<0.0001	0.98 (0.97, 0.99)	0.0002	0.98 (0.97, 1.00)	0.0092
GNRI < 92	0.95 (0.93, 0.97)	<0.0001	0.95 (0.93, 0.97)	<0.0001	0.94 (0.92, 0.97)	<0.0001
GNRI ≥ 92	0.99 (0.97, 1.00)	0.0131	0.99 (0.98, 1.00)	0.085	1.00 (0.98, 1.01)	0.5532

*Adjust model-I: age and gender*.

*Adjust model-II: age, gender, ethnicity, SPO_2_, HR, RR, SBP, DBP, ALT, AST, BUN, bicarbonate, glucose, potassium, creatinine, CLF, COPD, ARDS, AKI, STROKE, and malignancy*.

**Table 8:** Association between in-hospital mortality and GNRI (<92) observed using the multivariate logistic regression model.

**GNRI**	***N***	**Non-adjusted OR**	***P***	**Adjust-I OR**	***P***	**Adjust-II OR**	***P***
Total	739	0.95 (0.93, 0.97)	<0.0001	0.95 (0.93, 0.97)	<0.0001	0.94 (0.92, 0.97)	<0.0001
≤81	259	1.00 (ref)		1.00 (ref)		1.00 (ref)	
82–91	480	0.61 (0.44, 0.85)	0.0037	0.60 (0.43, 0.84)	0.0027	0.62 (0.42, 0.91)	0.0146

*Adjust model-I: age and gender*.

*Adjust model-II: age, gender, ethnicity, SPO_2_, heart rate, SYSBP, DIASBP, resp rate, ALT, AST, BUN, bicarbonate, sodium, chloride, potassium, creatinine, COPD, ARDS, AKI, STROKE, and CLF*.

## Discussion

This study found through multiple analyses that GNRI, which reflects the nutritional status of elderly patients, is closely related to the outcome of elderly patients with MODS during hospitalization. The univariate analysis showed that malnutrition (GNRI ≤ 98) significantly increases the risk of death during hospitalization in elderly patients with MODS (OR = 1.70, 95% CI: 1.39–2.07, *P* < 0.001). Further univariate analysis showed that moderate to severe malnutrition further increases the risk of death in elderly patients with MODS during hospitalization (OR = 1.83, 95% CI: 1.49–2.24, *P* < 0.001). For patients with MODS with GNRI < 92, GNRI is the protective factor, that is, a high GNRI results in low risk of death. Further subgroup analysis of 739 elderly patients with GNRI < 92 shows that the risk of death in elderly men is lower than that in elderly women. The results of this study suggest that mild malnutrition has no significant effect on the mortality risk of elderly patients with MODS during hospitalization, but elderly patients with MODS and moderate to severe malnutrition have high risk of death during hospitalization, and it is reasonable for patients to be evaluated upon admission to the ICU. The nutritional status of the baseline level is helpful in judging the treatment effect during hospitalization and suggests the necessity of nutritional support for elderly patients with MODS. The goal of nutritional support may be GNRI > 99, but accurate conclusions need further confirmation by prospective interventional studies.

At present, many countries enter an aging society, and the definition of old age has been raised from 60 to 65 years old. The number of elderly people (≥80 years old) increases significantly, leading to a continuous increase in the number of elderly patients admitted to ICUs (10). Elderly patients with MODS often have a variety of underlying diseases, and declining organ function with age, relatively weak physique, and weakened ability to compensate for severe diseases (11). When critically ill, the patient should be admitted to the ICU for monitoring and treatment. A considerable number of patients are at the end of their lives. Active invasive treatment (such as mechanical ventilation and various catheterization) cannot change the short-term outcome (12) and increases the suffering of patients and the financial burden of the family. At the same time, medical resources are becoming scarce. Thus, some critically ill patients with high therapeutic needs cannot get timely intensive care treatment (13). Therefore, the current medical field is gradually paying attention to research on the hospitalization outcome of elderly critically ill patients (14). Guidet et al. tried to reduce the 6-month mortality rate of elderly critically ill patients by promoting the admission of elderly critically ill patients to the ICU. Results found that increasing the admission of elderly patients to the ICU does not reduce the 6-month mortality rate, suggesting that increased intensive care treatment cannot improve the overall short-term prognosis of elderly critically ill patients (15). In terms of medical expenses, the study found that elderly and critically ill patients need medical resources, and the corresponding expenditures are significantly higher than those of other patients in other age groups. The mortality rate during hospitalization is highest in very elderly patients with severe illness followed by middle elderly patients (65–80 years) and lowest in patients under 65 years of age (16). Therefore, effective evaluation methods are needed to predict the death risk of elderly critically ill patients during hospitalization to provide relevant information to patients, family members, and doctors for deciding whether to admit patients into the ICU. Demiselle et al. analyzed the clinical data of 501 elderly critically ill patients and found that 108 (21.6%) patients died during hospitalization. Factors related to death during hospitalization include high acute illness scores, ICU admission diagnosis (including cardiac resuscitation, anxiety, and worry about the deterioration of the quality of life in patients' agents), and staying in the chronic disease management institution before entering the ICU (17). For very elderly patients (≥90 years), mechanical ventilation is a predictor of death during ICU hospitalization but not age (18). These studies are aimed at the entire group of elderly patients with severe illness, and few similar studies are aimed at elderly patients with MODS.

Nutrition plays an important role in the normal physiological functions of the human body and treatment of diseases especially in elderly patients (19, 20). Currently, a variety of nutritional status evaluation tools, including Nutritional Risk Screening 2002, Nutrition Risk in the Critically Ill, GNRI, are available (21–23). Among these tools, GNRI is a scoring tool specific for elderly patients. Clinical studies found that GNRI can predict the short-term prognosis of critically ill patients, such as those with heart failure, respiratory failure, and sepsis (6, 24, 25), and the long-term prognosis of patients with cancer (8). These studies showed that GNRI has a certain relationship with the prognosis of severely ill elderly patients, but most studies have small samples. Given the diversity of critically ill patients, prospective controlled studies are difficult to carry out. The MIMIC database provides a large amount of real-world data on elderly critically ill patients (26). After screening, the data of 2,456 elderly patients with MODS are included, and the sample size of our study has increased significantly compared with those in other studies. The analysis results further prove that GNRI can effectively predict the death risk of elderly patients with MODS during hospitalization. Nutritional status often reflects the actual situation of the body's intake of nutrients for a period. The intake and absorption of nutrients are related to many factors, including the patient's basic diseases, living habits, and treatment (27). Malnutrition often indicates that the patient is in an unoptimistic situation. When the disease progresses to a critical illness with MODS, the risk of death is significantly increased (28, 29). Therefore, based on previous research and our results, we believe that for elderly patients with MODS, the baseline GNRI can be calculated before admission to the ICU, and the short-term prognosis of the patients can be comprehensively evaluated on the basis of GNRI, thereby providing valuable information for further decision-making.

This study has certain limitations. The main reason is that the data of this study come from the MIMIC database. Even with its large inventory sample size, this study remains a retrospective analysis. As a result, some cases may be eliminated due to incomplete data. At the same time, some useful indicators are not tested or entered, which may affect the results of the analysis. Another limitation is that multivariate logistic regression equation was used to calculate the odds ratio (OR) of in-hospital deaths among different groups, but the real-world data may not be linear. For a reliable evidence, further multicenter registration studies, especially prospective cohort studies, are still needed.

## Conclusion

GNRI is related to the severity of illness in elderly patients with MODS. When GNRI < 92, moderate to severe malnutrition increases the risk of death in elderly patients with MODS during hospitalization. In clinical practice, whether elderly patients with MODS have malnutrition should be determined. For patients with malnutrition, reasonable supplementary nutrition should be given to avoid poor prognosis due to malnutrition.

## Data Availability Statement

The raw data supporting the conclusions of this article will be made available by the authors, without undue reservation.

## Ethics Statement

Ethical review and approval was not required for the study on human participants in accordance with the local legislation and institutional requirements. Written informed consent for participation was not required for this study in accordance with the national legislation and the institutional requirements. Written informed consent was not obtained from the individual(s) for the publication of any potentially identifiable images or data included in this article.

## Author Contributions

FZ contributed to the study initial design. ZM and TW performed the statistical analysis. XL, JC, PH, CL, HL, HK, and ZZ contributed to the data collection and data cleansing. ZM made the charts and drafted the manuscript. All authors read and approved the final manuscript.

## Funding

This study was supported by Medical Innovation Research (18CXZ026) and Medical Comprehensive Program (20BJZ27). The funding sources had no role in study design, data collection, data analysis, data interpretation, or writing of the report.

## Conflict of Interest

The authors declare that the research was conducted in the absence of any commercial or financial relationships that could be construed as a potential conflict of interest.

## Publisher's Note

All claims expressed in this article are solely those of the authors and do not necessarily represent those of their affiliated organizations, or those of the publisher, the editors and the reviewers. Any product that may be evaluated in this article, or claim that may be made by its manufacturer, is not guaranteed or endorsed by the publisher.

## References

[1] Angus DC. Admitting elderly patients to the intensive care Unit-Is it the right decision? JAMA. (2017) 318:1443–4. 10.1001/jama.2017.14535 28973429

[2] Awad A Bader-El-Den M McNicholas J Briggs J. Early hospital mortality prediction of intensive care unit patients using an ensemble learning approach. Int J Med Inform. (2017) 108:185–95. 10.1016/j.ijmedinf.2017.10.002 29132626

[3] Reyes JC Alonso JV Fonseca J Santos ML Jimenez ML Braniff J. Characteristics and mortality of elderly patients admitted to the Intensive Care Unit of a district hospital. Indian J Crit Care Med. (2016) 20:391–7. 10.4103/0972-5229.186219 27555692 PMC4968060

[4] Shpata V Ohri I Nurka T Prendushi X. The prevalence and consequences of malnutrition risk in elderly Albanian intensive care unit patients. Clin Interv Aging. (2015) 10:481–6. 10.2147/CIA.S77042 25733824 PMC4337415

[5] Corish CA Bardon LA. Malnutrition in older adults: Screening and determinants. Proc Nutr Soc. (2019) 78:372–9. 10.1017/S0029665118002628 30501651

[6] Li H Cen K Sun W Feng B. Prognostic value of geriatric nutritional risk index in elderly patients with heart failure: a meta-analysis. Aging Clin Exp Res. (2021) 33:1477–86. 10.1007/s40520-020-01656-3 32766928

[7] Wei L Xie H Li J Li R Chen W Huang L. The prognostic value of geriatric nutritional risk index in elderly patients with severe community-acquired pneumonia: a retrospective study. Medicine. (2020) 99:e22217. 10.1097/MD.0000000000022217 32925799 PMC7489621

[8] Lidoriki I Schizas D Frountzas M Machairas N Prodromidou A Kapelouzou A. GNRI as a prognostic factor for outcomes in cancer patients: a systematic review of the literature. Nutr Cancer. (2021) 73:391–403. 10.1080/01635581.2020.1756350 32321298

[9] Chen H Zhu Z Zhao C Guo Y Chen D Wei Y. Central venous pressure measurement is associated with improved outcomes in septic patients: an analysis of the MIMIC-III database. Crit Care. (2020) 24:433. 10.1186/s13054-020-03109-9 32665010 PMC7358999

[10] Chin-Yee N D'Egidio G Thavorn K Heyland D Kyeremanteng K. Cost analysis of the very elderly admitted to intensive care units. Crit Care. (2017) 21:109. 10.1186/s13054-017-1689-y 28506243 PMC5433056

[11] Bagshaw SM Webb SA Delaney A George C Pilcher D Hart GK. Very old patients admitted to intensive care in Australia and New Zealand: a multi-centre cohort analysis. Crit Care. (2009) 13:R45. 10.1186/cc7768 19335921 PMC2689489

[12] Nguyen YL Angus DC Boumendil A Guidet B. The challenge of admitting the very elderly to intensive care. Ann Intensive Care. (2011) 1:29. 10.1186/2110-5820-1-29 21906383 PMC3224497

[13] Flaatten H de Lange DW Artigas A Bin D Moreno R Christensen S. The status of intensive care medicine research and a future agenda for very old patients in the ICU. Intensive Care Med. (2017) 43:1319–28. 10.1007/s00134-017-4718-z 28238055

[14] Leblanc G Boumendil A Guidet B. Ten things to know about critically ill elderly patients. Intensive Care Med. (2017) 43:217–9. 10.1007/s00134-016-4477-2 27492269

[15] Guidet B Leblanc G Simon T Woimant M Quenot JP Ganansia O. Effect of systematic intensive care unit triage on long-term mortality among critically ill elderly patients in france: a randomized clinical trial. JAMA. (2017) 318:1450–9. 10.1001/jama.2017.13889 28973065 PMC5710364

[16] Haas L van Beusekom I van Dijk D Hamaker ME Bakhshi-Raiez F de Lange DW. Healthcare-related costs in very elderly intensive care patients. Intensive Care Med. (2018) 44:1896–903. 10.1007/s00134-018-5381-8 30255319

[17] Demiselle J Duval G Hamel JF Renault A Bodet-Contentin L Martin-Lefevre L. Determinants of hospital and one-year mortality among older patients admitted to intensive care units: Results from the multicentric SENIOREA cohort. Ann Intensive Care. (2021) 11:35. 10.1186/s13613-021-00804-w 33595733 PMC7889762

[18] Le Borgne P Maestraggi Q Couraud S Lefebvre F Herbrecht JE Boivin A. Critically ill elderly patients (>/= 90 years): Clinical characteristics, outcome and financial implications. PLoS ONE. (2018) 13:e198360. 10.1371/journal.pone.0198360 29856809 PMC5983531

[19] Mastronuzzi T Grattagliano I. Nutrition as a health determinant in elderly patients. Curr Med Chem. (2019) 26:3652–61. 10.2174/0929867324666170523125806 28545376

[20] Barkoukis H. Nutrition recommendations in elderly and aging. Med Clin North Am. (2016) 100:1237–50. 10.1016/j.mcna.2016.06.006 27745592

[21] Coruja MK Cobalchini Y Wentzel C Fink J. Nutrition risk screening in intensive care units: agreement between NUTRIC and NRS 2002 tools. Nutr Clin Pract. (2020) 35:567–71. 10.1002/ncp.10419 31602679

[22] Kondrup J. Nutrition risk screening in the ICU. Curr Opin Clin Nutr Metab Care. (2019) 22:159–61. 10.1097/MCO.0000000000000551 30601175

[23] Lee JS Jeong KY Ko SH. Usefulness of the Geriatric Nutritional Risk Index to predict the severity of cholecystitis among older patients in the emergency department. Geriatr Gerontol Int. (2020) 20:455–60. 10.1111/ggi.13900 32147936

[24] Yenibertiz D Cirik MO. The comparison of GNRI and other nutritional indexes on short-term survival in geriatric patients treated for respiratory failure. Aging Clin Exp Res. (2021) 33:611–7. 10.1007/s40520-020-01740-8 33130989

[25] Lee JS Choi HS Ko YG Yun DH. Performance of the Geriatric Nutritional Risk Index in predicting 28-day hospital mortality in older adult patients with sepsis. Clin Nutr. (2013) 32:843–8. 10.1016/j.clnu.2013.01.007 23391456

[26] Zhang Y Zheng Q Dai X Xu X Ma L. Overweight is associated with better one-year survival in elderly patients after cardiac surgery: A retrospective analysis of the MIMIC-III database. J Thorac Dis. (2021) 13:562–74. 10.21037/jtd-20-2824 33717529 PMC7947548

[27] Wells JC Sawaya AL Wibaek R Mwangome M Poullas MS Yajnik CS. The double burden of malnutrition: Aetiological pathways and consequences for health. Lancet. (2020) 395:75–88. 10.1016/S0140-6736(19)32472-9 31852605 PMC7613491

[28] Schneider SM Correia M. Epidemiology of weight loss, malnutrition and sarcopenia: A transatlantic view. Nutrition. (2020) 69:110581. 10.1016/j.nut.2019.110581 31622908

[29] Leiva BE Badia TM Virgili CN Elguezabal SG Faz MC Herrero MI. Hospital malnutrition screening at admission: Malnutrition increases mortality and length of stay. Nutr Hosp. (2017) 34:907–13. 10.20960/nh.657 29095016

